# Changes in muscle cross-sectional area and functional outcomes after hip arthroscopy for femoroacetabular impingement: A comparison between the operated and asymptomatic contralateral hip

**DOI:** 10.1051/sicotj/2026053

**Published:** 2026-08-18

**Authors:** Yasin Erdoğan, Mehtap Oktay, Şahan Güven, Enejd Veizi, Hilmi Alkan, Kemal Şibar, Ahmet Firat

**Affiliations:** 1 Ankara Etlik City Hospital, Department of Orthopaedics and Traumatology Ankara Turkey; 2 Ankara Etlik City Hospital, Department of Radiology Ankara Turkey; 3 Ankara Bilkent City Hospital, Department of Orthopaedics and Traumatology Ankara Turkey; 4 Sultan 2. Abdulhamid Han Training and Research Hospital, Department of Orthopaedics and Traumatology İstanbul Turkey; 5 İstinye University, Vm Medical Park Hospital, Department of Orthopaedics and Traumatology Ankara Turkey

**Keywords:** Femoroacetabular impingement, Hip arthroscopy, Muscle cross-sectional area, Magnetic resonance imaging, Functional outcomes

## Abstract

*Introduction:* The effect of hip arthroscopy on peri-hip muscle morphology in patients with femoroacetabular impingement syndrome (FAI) remains unclear. Understanding postoperative muscle recovery and its relationship with functional outcomes may help optimize rehabilitation strategies. This study aimed to evaluate preoperative and 1-year postoperative changes in peri-hip muscle cross-sectional areas (CSAs) on both the operated and asymptomatic contralateral sides in patients with FAI undergoing hip arthroscopy and to assess their association with functional outcomes. *Methods:* This retrospective study included 66 patients who underwent hip arthroscopy for FAI, including femoroplasty, acetabuloplasty, labral repair, and complete capsular closure performed by a single surgeon. Axial T1-weighted pelvic magnetic resonance imaging scans obtained preoperatively and at 1-year postoperatively were analyzed. Bilateral CSAs of the gluteal, iliopsoas, iliocapsularis, quadriceps, and adductor muscle groups were measured. Functional outcomes were evaluated using the modified Harris Hip Score, Non-Arthritic Hip Score, Hip Outcome Score–Activities of Daily Living, Hip Outcome Score–Sports-Specific Subscale, Hip disability and Osteoarthritis Outcome Score, and Visual Analog Scale. *Results:* At the 1-year follow-up, the gluteus maximus and rectus femoris CSAs on the operated side remained significantly smaller than those on the asymptomatic side. No significant postoperative increase in gluteus maximus CSA was observed, whereas the vastus medialis and rectus femoris demonstrated significant postoperative increases. Most other peri-hip muscles showed comparable pre- and postoperative CSAs between sides. All functional outcome scores improved significantly postoperatively, while Visual Analog Scale scores decreased. Postoperative gluteus maximus and iliocapsularis CSAs were positively correlated with postoperative modified Harris Hip Score values. *Conclusions:* Although most peri-hip muscle CSAs do not fully recover to asymptomatic side levels at 1 year following hip arthroscopy for FAI, improvements in specific muscle groups are associated with better functional outcomes. These findings underscore the importance of targeted postoperative rehabilitation focusing on key stabilizing muscles, particularly the gluteus maximus and iliocapsularis.

## Introduction

Femoroacetabular impingement syndrome (FAI) is a major cause of hip pain in young adults and an important contributor to early-stage osteoarthritis, resulting from repetitive minor trauma caused by abnormal bony morphology that leads to chondrolabral injury [1–4]. Arthroscopic treatment of femoroacetabular impingement has been reported to result in favorable rates of return to sport as well as satisfactory long-term clinical and functional outcomes [5–7].

Muscle weakness associated with knee and hip osteoarthritis, along with MRI-based studies evaluating muscle cross-sectional area (CSA), has been widely reported in the literature [8–10]. Several studies have demonstrated muscle weakness in patients with femoroacetabular impingement compared with healthy control groups, and others have evaluated the relationship between preoperative muscle CSA and postoperative clinical outcomes [11, 12]. Following hip arthroscopy, delayed weight bearing and postoperative restrictions on certain movements may lead to muscle atrophy in the affected muscle groups [13].

The studies comparing preoperative and postoperative muscle CSA on axial MRI following hip arthroscopy are limited, and the range of hip functional outcome scores used in these studies is also restricted [14, 15]. On the other hand, data comparing the operated hip with the postoperative asymptomatic contralateral hip in patients with femoroacetabular impingement are scarce, and the surgical procedures performed in these patients are heterogeneous.

The purpose of this study was to evaluate preoperative and 1-year postoperative muscle CSAs on both the operated and asymptomatic sides in a homogeneous patient cohort who underwent femoroplasty, acetabuloplasty, labral repair, and complete capsular closure, and to compare these findings with functional outcome scores. This study hypothesized that hip arthroscopy would lead to increases in muscle area and improvements in functional scores at 1-year postoperatively.

## Material and methods

### Study design and participants

After obtaining institutional review board approval, patients who underwent femoroplasty, acetabuloplasty, labral repair, and complete capsular closure for the diagnosis of combined femoroacetabular impingement syndrome [1] between 2022 and 2024 were identified. All procedures were performed by a single senior hip surgeon. Patients who underwent pelvic MRI at the 1-year postoperative follow-up for any indication, had no history of surgery on the contralateral hip, and were asymptomatic on the contralateral side were included in the study. Patients were excluded if they had incomplete or externally performed MRI data, underwent revision hip arthroscopy, had a Tönnis grade greater than 1, had bilateral femoroacetabular impingement, had a history of contralateral hip surgery or symptoms, had undergone any peri-hip tendon repair procedures, or had iliopsoas release performed in addition to femoroacetabular impingement surgery.

### Outcome measures

For all included patients, demographic data including age, sex, and laterality were recorded. Muscle CSA measurements were obtained on axial T1-weighted MRI sequences for both the operated and contralateral hips, and functional outcome scores were evaluated. On axial images, CSAs of the gluteus maximus (G. maximus), gluteus medius/minimus complex (G. medius/minimus), rectus femoris (R. femoris), iliocapsularis, iliopsoas, vastus lateralis (V. lateralis), vastus medialis (V. medialis), vastus intermedius (V. intermedius), adductor longus (A. longus), adductor magnus (A. magnus), and adductor brevis (A. brevis) muscles were measured.

Functional outcomes were assessed preoperatively and at the 1-year follow-up using the modified Harris Hip Score (mHHS), Non-Arthritic Hip Score (NAHS), Hip Outcome Score–Activities of Daily Living (HOS-ADL), Hip Outcome Score–Sports-Specific Subscale (HOS-SSS), and the Visual Analog Scale (VAS).

**Figure 1:**
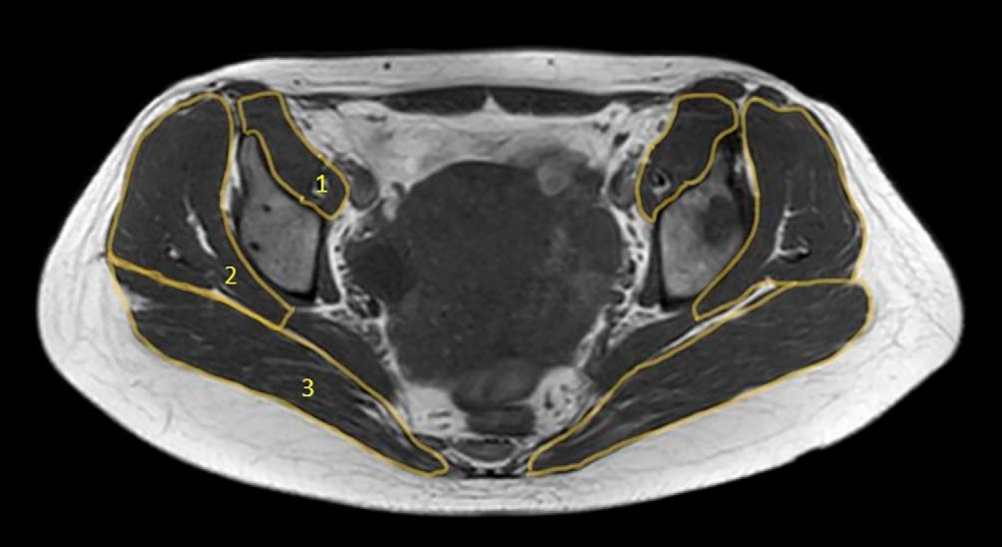
Cross-sectional area 1 – iliopsoas; 2 – gluteus medius/minimus complex, 3 – gluteus maximus.

**Figure 2:**
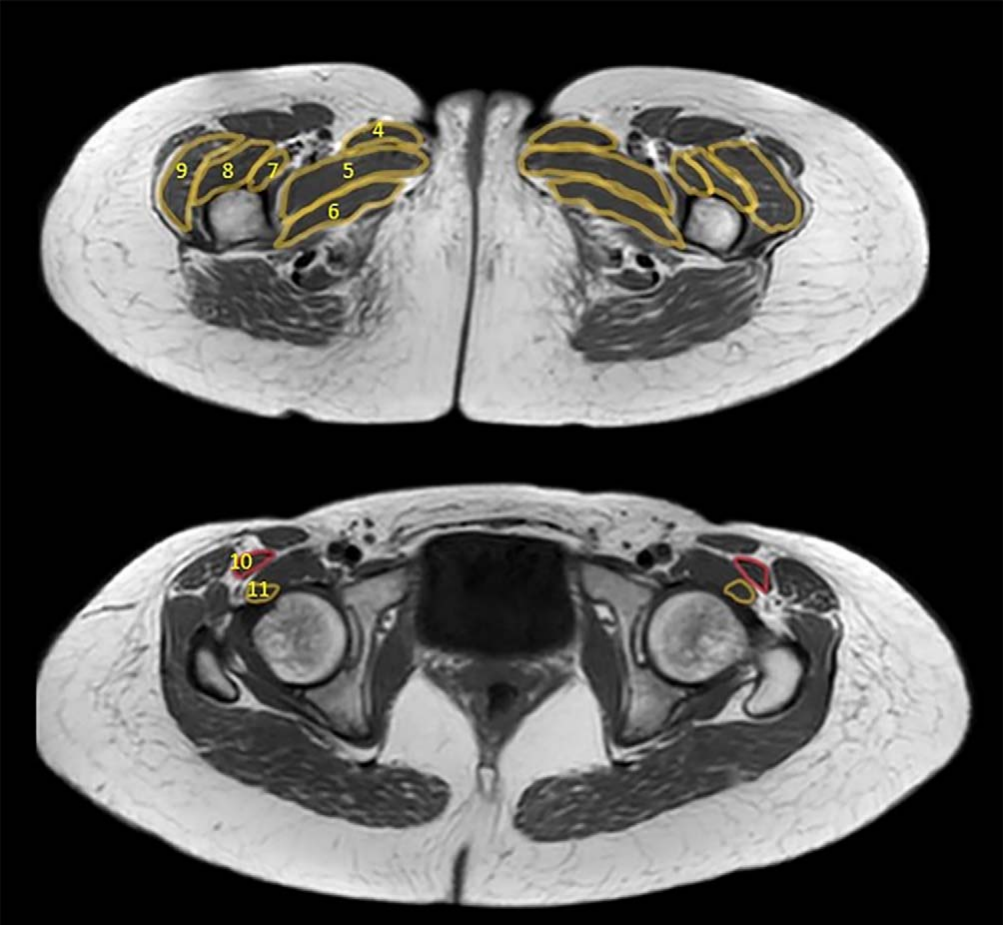
Cross-sectional area 4 – adductor magnus, 5 – adductor longus, 6 – adductor brevis, 7 – vastus medialis, 8 – vastus intermedius, 9 – vastus lateralis, 10 – rectus femoris, 11 – iliocapsularis.

The hip MRI protocol consisted of a complete pelvic MRI examination, including both hip joints. All MRI measurements were performed using a 3.0-Tesla scanner (Philips Ingenia Elition, Netherlands) with a standard hip coil. The imaging protocol included coronal non–fat-suppressed T1-weighted and fat-suppressed proton density (PD)-weighted sequences, as well as axial non–fat-suppressed T1-weighted and PD-weighted sequences, acquired with a slice thickness of 4 mm. To provide optimal anatomical detail, muscle CSA measurements were obtained on axial T1-weighted images. All measurements were performed using a Picture Archiving and Communication System (PACS). In accordance with previously published studies [12], regions of interest (ROIs) were manually delineated bilaterally around each muscle to quantify muscle CSA. All measurements were performed by a single radiologist with expertise in musculoskeletal imaging. To ensure interpatient standardization of pelvic and thigh muscle measurements, three distinct anatomical levels were defined. Measurements of the G. maximus, G. medius/G. minimus complex, and iliopsoas were obtained at the level of the inferior border of the ilium. The iliocapsularis and R. femoris muscles were measured at the level where the femoral head appeared most spherical. Measurements of the V. lateralis, V. intermedius, V. medialis, A. magnus, A. longus, and A. brevis muscles were obtained at the most proximal portion of the femoral diaphysis (Figures 1 and 2).

### Arthroscopic surgery

All patients were positioned supine on a traction table under general anesthesia. To protect the perineal region, a soft cylindrical perineal post with a diameter of 30 cm was used in all cases. The hip joint was initially accessed through a standard anterolateral (AL) portal to evaluate central compartment pathology, followed by establishment of a mid-anterior portal. After interportal capsulotomy [16], a single traction suture was placed through the proximal portion of the capsule. Following the widening of the capsulolabral interval, the existing pincer lesion was first exposed. Based on preoperative planning, acetabular rim trimming was performed, and the labrum was subsequently repaired using suture anchors through the distal anterolateral (DALA) portal. Traction was then released, and a T-shaped capsulotomy was performed to the extent necessary to visualize the cam lesion and access the peripheral compartment. Traction sutures were placed along the edges of the capsulotomy [17]. Under fluoroscopic guidance and according to preoperative planning, the cam lesion was resected until adequate femoral head–neck offset restoration was achieved. Finally, a routine complete capsular closure was performed, and the procedure was concluded.

### Postoperative rehabilitation

Postoperatively, patients were allowed partial weight bearing with crutches for 4 weeks. Isometric exercises for the gluteal, quadriceps, and adductor muscle groups were initiated immediately. During the first 2 weeks, hip flexion was limited to 100°, and external rotation was not permitted with the hip in the neutral position. At 90° of hip flexion, internal rotation was limited to 10° and external rotation to 30°. Excessive hip extension was restricted during the first postoperative month. Rehabilitation intensity during the initial 4 weeks was guided by pain tolerance, and highly painful rehabilitation was avoided. After the fourth postoperative week, weight bearing was gradually increased, with full weight bearing targeted by week 6. Hip range of motion goals were advanced to 120° of flexion and 40° of external rotation. Active strengthening exercises were initiated after week 4, focusing on the gluteal, quadriceps, hamstring, and adductor muscle groups. From week 6 onward, stair climbing, balance training, and dynamic core exercises were introduced. Low-speed jogging was permitted after week 12, followed by progression to running on variable surfaces after week 16. Plyometric exercises, including single- and double-leg hopping and agility drills, were initiated after week 20. Return to unrestricted daily activities was targeted after week 32. Return to sports was permitted after muscle strength reached at least 90% of the contralateral side.

### Statistical analysis

Statistical analyses were performed using SPSS software (version 27.0; IBM Corp., Armonk, NY, USA). Descriptive statistics were presented as mean ± standard deviation, median (minimum–maximum), frequency, and percentage, as appropriate. The normality of data distribution was assessed using the Kolmogorov–Smirnov and Shapiro–Wilk tests. As the data were not normally distributed, the Wilcoxon signed-rank test was used for repeated-measures comparisons. Correlations between muscle area measurements and functional outcome scores were evaluated using Spearman’s rank correlation analysis. A *p*-value of <0.05 was considered statistically significant.

A priori sample size calculation was performed using G*Power software (version 3.1.9.7). The primary outcome of the study was defined as the difference in muscle CSA measurements between the operated and contralateral asymptomatic hips on magnetic resonance imaging (MRI). Assuming a medium effect size (Cohen’s *d* = 0.50), a type I error rate of 5% (*α* = 0.05), and a statistical power of 80% (1 − *β* = 0.80), the minimum required sample size was calculated as 34 patients. Considering the possibility of dropout and incomplete data during follow-up, the target sample size was increased by 20%, and at least 42 patients were planned for inclusion.

## Results

**Figure 3:**
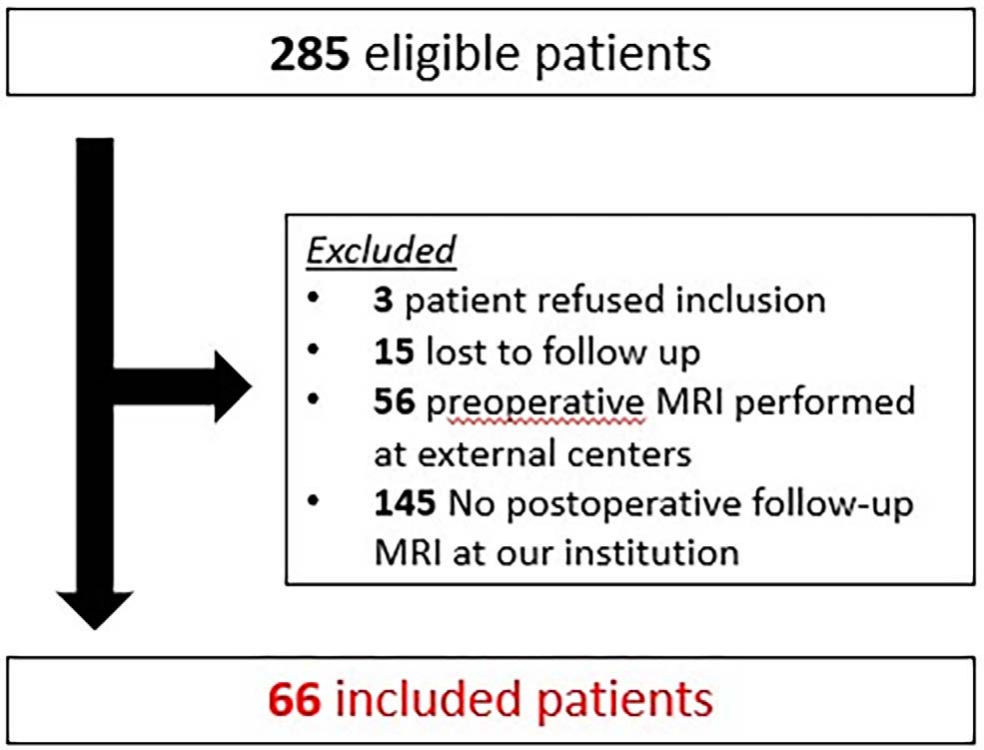
Flowchart of the patient selection.

Between the study dates, a total of 285 patients underwent hip arthroscopy for the diagnosis of femoroacetabular impingement. Of these, 66 patients with complete follow-up and available pelvic MRI images obtained both preoperatively and at the 1-year postoperative follow-up were included in the study (Figure 3).

The mean age was 34.8 years (SD: ±6.8). Thirty-six (54.5%) of the patients underwent surgery on the right side, and 30 (45.5%) on the left side. The mean time to postoperative MRI was 12.2 ± 0.4 months, with a median of 12 months (range, 12–13 months) (Table 1).

**Table 1:** Baseline demographic characteristics and muscle cross-sectional areas.

		Min–Max			Median	Mean ± SD/*n*-%		
Age		24.0	–	51.0	34.0	34.8	±	6.8
Gender	Male					34		51.5%
	Female					32		48.5%
Side	Right					36		54.5%
	Left					30		45.5%
Gluteus maximus (mm^2^)								
Operated side		3060.0	–	6400.0	4900.0	4706.5	±	829.2
Asymptomatic side		3100.0	–	6900.0	4900.0	4753.9	±	885.5
Gluteus medius/Minimus complex (mm^2^)								
Operated side		2350.0	–	4640.0	2910.0	3089.7	±	518.0
Asymptomatic side		2200.0	–	4670.0	2920.0	3117.3	±	541.9
Rectus femoris (mm^2^)								
Operated side		100.0	–	160.0	120.0	122.6	±	12.7
Asymptomatic side		110.0	–	170.0	130.0	130.3	±	14.4
Iliocapsularis (mm^2^)								
Operated side		110.0	–	180.0	140.0	138.6	±	18.9
Asymptomatic side		110.0	–	180.0	140.0	139.4	±	14.9
Iliopsoas (mm^2^)								
Operated side		900.0	–	2120.0	1410.0	1364.8	±	273.1
Asymptomatic side		960.0	–	2100.0	1400.0	1363.5	±	276.3
Vastus lateralis (mm^2^)								
Operated side		920.0	–	2030.0	1150.0	1248.9	±	285.4
Asymptomatic side		900.0	–	1700.0	1240.0	1240.0	±	222.4
Vastus medialis (mm^2^)								
Operated side		260.0	–	790.0	420.0	438.3	±	105.6
Asymptomatic side		270.0	–	680.0	450.0	458.5	±	110.2
Vastus intermedius (mm^2^)								
Operated side		250.0	–	940.0	430.0	450.5	±	139.7
Asymptomatic side		270.0	–	1000.0	440.0	453.9	±	138.4
Adductor longus (mm^2^)								
Operated side		800.0	–	2060.0	1080.0	1127.3	±	250.6
Asymptomatic side		750.0	–	1830.0	1100.0	1118.2	±	212.9
Adductor magnus (mm^2^)								
Operated side		450.0	–	980.0	690.0	688.8	±	152.4
Asymptomatic side		440.0	–	960.0	650.0	664.4	±	142.4
Adductor brevis (mm^2^)								
Operated side		700.0	–	1400.0	1020.0	1017.0	±	162.5
Asymptomatic side		700.0	–	1400.0	1000.0	1017.3	±	166.8

mm^2^: square millimeter.

On the operated side, both the preoperative and postoperative G. maximus values were significantly lower than those of the contralateral side, although the effect size was weak (Cohen’s *d* = 0.06 and *d* = 0.09, respectively). On the operated side, no significant change was observed in postoperative G. maximus values compared with preoperative measurements. Similarly, no significant difference was found between preoperative and postoperative G. maximus values on the contralateral side (Table 2).

**Table 2:** Preoperative and postoperative muscle cross-sectional areas of the operated and asymptomatic sides.

	Operated side				Asymptomatic side				*p*
	Mean ± SD			Median	Mean ± SD			Median	
Gluteus maximus (mm^2^)									
Preoperative	4706.5	±	829.2	4900	4753.9	±	885.5	4900	*0*.*007*^W^
Postoperative	4717	±	818.5	4850	4794.5	±	857.1	4900	*0*.*000*^W^
Preoperative/Postoperative difference	10.5	±	251.8	50	40.6	±	195.2	40	0.656^W^
*Intra-group difference p*	0.521^W^				0.094^W^				
Gluteus medius/Minimus complex (mm^2^)									
Preoperative	3089.7	±	518	2910	3117.3	±	541.9	2920	0.098^W^
Postoperative	3063	±	460	3090	3101.2	±	468.6	3050	0.086^W^
Preoperative/Postoperative difference	−26.7	±	315.4	−30.0	−16.1	±	325	0	0.988^W^
*Intra-group difference p*	0.124^W^				0.611^W^				
Rectus femoris (mm^2^)									
Preoperative	122.6	±	12.7	120	130.3	±	14.4	130.0	*0*.*000*^W^
Postoperative	125.8	±	14	125	135.6	±	21.1	130	*0*.*003*^W^
Preoperative/Postoperative difference	3.2	±	13.4	0	5.3	±	22.9	0	0.679^W^
*Intra-group difference p*	*0*.*030*^W^				0.122^W^				
Iliocapsularis (mm^2^)									
Preoperative	138.6	±	18.9	140	139.4	±	14.9	140	0.442^W^
Postoperative	140	±	16.9	140	153.2	±	21.8	150	*0*.*000*^W^
Preoperative/Postoperative difference	1.4	±	19.5	0	13.8	±	22.8	10	*0*.*004*^W^
*Intra-group difference p*	0.745^W^				*0*.*000*^W^				
Iliopsoas (mm^2^)									
Preoperative	1364.8	±	273.1	1410	1363.5	±	276.3	1400	0.923^W^
Postoperative	1399.4	±	265	1440	1404.2	±	247	1500	0.329^W^
Preoperative/Postoperative difference	34.5	±	97.2	40	40.8	±	103.6	50	0.498^W^
*Intra-group difference p*	*0*.*007*^W^				*0*.*002*^W^				
Vastus lateralis (mm^2^)									
Preoperative	1248.9	±	285.4	1150	1240	±	222.4	1240	0.939^W^
Postoperative	1284.8	±	286	1230	1303.3	±	334	1200	0.392^W^
Preoperative/Postoperative difference	35.9	±	165	60	63.3	±	222.9	60	0.877^W^
*Intra-group difference p*	*0*.*020*^W^				*0*.*023*^W^				
Vastus medialis (mm^2^)									
Preoperative	438.3	±	105.6	420	458.5	±	110.2	450	*0*.*043*^W^
Postoperative	469.8	±	97.7	470	473.5	±	99.4	460	0.239^W^
Preoperative/Postoperative difference	31.5	±	65.3	20	15	±	97.2	30	0.157^W^
*Intra-group difference p*	*0*.*000*^W^				0.231^W^				
Vastus intermedius (mm^2^)									
Preoperative	450.5	±	139.7	430	453.9	±	138.4	440	0.266^W^
Postoperative	466.7	±	128.7	470	500	±	131.6	490	*0*.*000*^W^
Preoperative/Postoperative difference	16.2	±	85	30	46.1	±	79.1	45	*0*.*007*^W^
*Intra-group difference p*	0.053^W^				*0*.*000*^W^				
Adductor longus (mm^2^)									
Preoperative	1127.3	±	250.6	1080	1118.2	±	212.9	1100	0.915
Postoperative	1147.6	±	205.5	1100	1123.6	±	162.2	1100	0.482
Preoperative/Postoperative difference	20.3	±	141.7	40	5.5	±	127.1	20	0.297
*Intra-group difference p*	0.117^W^				0.215^W^				
*Adductor magnus (mm^2^)*									
Preoperative	688.8	±	152.4	690	664.4	±	142.4	650	0.254^W^
Postoperative	697	±	144.3	650	632	±	114.5	620	*0*.*001*^W^
Preoperative/Postoperative difference	8.2	±	166.8	20	−32.4	±	156.6	−40.0	0.100^W^
*Intra-group difference p*	0.508^W^				0.157^W^				
Adductor brevis (mm^2^)									
Preoperative	1017	±	162.5	1020	1017.3	±	166.8	1000	0.689^W^
Postoperative	1019.4	±	150.3	1000	1019.2	±	153	1000	0.641^W^
Preoperative/Postoperative difference	2.4	±	114.8	0	2	±	119.7	20	0.781^W^
*Intra-group difference p*	0.596^W^				0.422^W^				

w: Wilcoxon test.

No significant differences in the G. medius/G. minimus complex CSA were observed between the operated and contralateral sides in either the preoperative or postoperative assessments. Additionally, postoperative G. medius/G. minimus complex CSA did not demonstrate a significant change compared with preoperative values on either side. The magnitude of preoperative-to-postoperative change in G. medius/G. minimus complex CSA was similar between the two sides (Table 2).

On the operated side, both the preoperative and postoperative CSAs of the R. femoris muscle were significantly lower than those on the contralateral side, with moderate effect sizes (Cohen’s *d* = 0.57 and *d* = 0.55, respectively) On the operated side, the CSA of the R. femoris muscle showed a significant increase postoperatively compared to the preoperative measurements, although the effect size was small (Cohen’s *d* = 0.24). On the contralateral side, postoperative R. femoris CSA increased relative to preoperative values. However, this change did not reach statistical significance. The magnitude of preoperative-to-postoperative change in R. femoris CSA did not differ significantly between the two sides (Table 2).

No significant difference in preoperative iliocapsularis CSA was observed between the operated and contralateral sides. On the operated side, postoperative iliocapsularis CSA did not demonstrate a significant change compared with preoperative values. In contrast, on the contralateral side, postoperative iliocapsularis CSA showed a significant increase relative to preoperative measurements. The magnitude of preoperative-to-postoperative change in iliocapsularis CSA was significantly greater on the contralateral side compared with the operated side (Table 2).

Preoperative and postoperative iliopsoas CSAs were similar between the operated and contralateral sides. On both sides, postoperative iliopsoas CSA demonstrated a significant increase compared with preoperative values. The magnitude of preoperative-to-postoperative change in iliopsoas CSA was comparable between the two sides (Table 2).

On both sides, preoperative and postoperative V. lateralis CSAs were comparable. Postoperative V. lateralis CSA demonstrated a significant increase compared with preoperative values on both sides. The magnitude of preoperative-to-postoperative change in V. lateralis CSA was similar between the operated and contralateral sides (Table 2).

On the operated side, the preoperative CSA of the V. medialis muscle was significantly lower than that of the contralateral side, although the effect size was small (Cohen’s *d* = 0.19). Postoperatively, the CSAs of the V. medialis muscle were comparable between the two sides. On the operated side, the postoperative CSA of the V. medialis muscle showed a significant increase compared to the preoperative values, with a moderate effect size (Cohen’s *d* = 0.31), whereas no significant postoperative increase was observed on the contralateral side. The magnitude of preoperative-to-postoperative change in V. medialis CSA was similar between the operated and contralateral sides (Table 2).

The sides were comparable with respect to preoperative V. intermedius CSA. Postoperatively, V. intermedius CSA was significantly lower on the operated side compared with the contralateral side. On the operated side, postoperative V. intermedius CSA was similar to preoperative values. In contrast, on the contralateral side, postoperative V. intermedius CSA demonstrated a significant increase compared with preoperative measurements. The magnitude of preoperative to postoperative change in V. intermedius CSA was significantly greater on the contralateral side than on the operated side (Table 2).

Preoperative A. magnus CSA was similar between the operated and contralateral sides. Postoperatively, the A. magnus CSA was significantly greater on the operated side compared with the contralateral side. On both sides, postoperative A. magnus CSAs were comparable to preoperative values. The magnitude of preoperative-to-postoperative change in A. magnus CSA was similar between the two sides (Table 2).

No significant differences in preoperative or postoperative A. longus CSA were observed between the affected and unaffected sides. On the affected side, postoperative A. longus CSA did not demonstrate a significant change compared with preoperative values. Similarly, no significant postoperative change in A. longus CSA was observed on the unaffected side. The magnitude of preoperative-to-postoperative change in A. longus CSA did not differ significantly between the affected and unaffected sides (Table 2).

Preoperative and postoperative CSAs of the A. longus and A. brevis muscles were similar between the operated and contralateral sides. On both sides, postoperative CSAs of the A. longus and A. brevis muscles were comparable. Additionally, the magnitude of preoperative-to-postoperative change in A. longus and A. brevis CSAs was similar on both sides (Table 2).

Significant improvements were observed in postoperative mHHS, NAHS, HOS-SSS, HOS-ADL, and HOOS scores, while a significant reduction was noted in VAS scores (Table 3).

**Table 3:** Patient-reported outcome of the operated side.

	Preoperative				Postoperative				*p*
	Mean ± SD			Median	Mean. ± `			Median	
mHHS	60.5	±	5.4	61.5	79.9	±	5.3	80.0	*0.000* ^W^
NAHS	45.4	±	5.5	43.0	76.1	±	3.4	77.0	*0.000* ^W^
HOS-SSS	51.1	±	7.3	50.3	71.8	±	9.6	70.6	*0.000* ^W^
HOS-ADL	59.5	±	7.1	56.6	80.1	±	5.5	79.3	*0.000* ^W^
HOOS	52.0	±	22.2	53.8	76.3	±	12.5	79.6	*0.000* ^W^
VAS	5.6	±	1.0	6.0	2.5	±	0.6	2.5	*0.000* ^W^

w: Wilcoxon test, mHHS: modified Harris Hip Score, NAHS: Non-Arthritic Hip Score, HOS-ADL: Hip Outcome Score–Activities of Daily Living, HOS-SSS: Hip Outcome Score–Sports-Specific Subscale, VAS: Visual Analog Scale.

A positive correlation was observed between postoperative G. maximus muscle area and postoperative mHHS scores (*ρ* = 0.34, *p* = 0.006). No significant association was identified between changes in the G. medius/G. minimus muscle complex area and VAS scores. A positive correlation was observed between postoperative G. medius/G. minimus muscle area and preoperative HOS-SSS and HOOS scores (*ρ* = 0.32, *p* = 0.009 for both). The postoperative iliopsoas muscle area was also found to be correlated with the preoperative HOS-SSS and HOOS scores (*ρ* = 0.28, *p* = 0.021 and *ρ* = 0.26, *p* = 0.036, respectively). Additionally, postoperative iliocapsularis muscle area was positively correlated with postoperative mHHS scores (*ρ* = 0.29, *p* = 0.019). Furthermore, an increase in A. magnus muscle area showed a weak correlation with postoperative HOOS scores (*ρ* = 0.26, *p* = 0.035). These findings indicate relationships between muscle CSA parameters and functional outcome scores; however, they should not be interpreted as evidence of a direct causal relationship.

## Discussion

The principal finding of the present study is that the CSAs of the gluteal and R. femoris muscles on the operated side were smaller than those of the preoperative asymptomatic side and did not demonstrate a significant increase at the 1-year postoperative follow-up. In contrast, although the CSAs of the V. medialis and R. femoris muscles were also reduced on the operated side compared with the preoperative asymptomatic side, both muscles exhibited a significant increase on the operated side at 1-year postoperatively. Although the effect sizes of the muscle area changes were not very large, they were statistically significant. Additionally, postoperative G. maximus and iliocapsularis muscle areas were found to be potentially associated with the mHHS, which may suggest a relationship between periarticular muscle morphology and clinical outcomes. As hypothesized, significant improvement in functional outcome scores was observed at the 1-year follow-up; however, contrary to our hypothesis, most muscle CSAs did not reach the levels of the asymptomatic contralateral side at 1 year postoperatively.

In a study comparing preoperative muscle CSAs between symptomatic and asymptomatic sides, the CSAs of the G. maximus, G. minimus, and R. femoris muscles were reported to be lower on the symptomatic side; however, postoperative muscle CSAs were not evaluated, and therefore no conclusions could be drawn regarding postoperative changes [12]. In a study by Yang et al., including 51 patients with a mean follow-up of 26 months, significant increases in the CSAs of the G. maximus and G. minimus muscles were observed compared with preoperative values, whereas no significant increases were noted in the G. medius or other muscle groups [14]. In another study, a reduction in the CSAs of the G. medius and G. minimus muscles was identified on the symptomatic side compared with the asymptomatic side [18]. In a study evaluating muscle CSA changes within the first postoperative year, a decrease in the CSAs of the G. maximus and the G. medius/G. minimus complex was observed at the 6-month follow-up [15]. At the 1-year follow-up, the G. maximus CSA returned to preoperative levels, whereas the G. medius/G. minimus complex did not reach preoperative values [15]. In this study, the G. maximus muscle CSA returned to preoperative levels at the 1-year postoperative follow-up. However, it remained lower than that of the asymptomatic contralateral side. The persistence of lower G. maximus CSA both preoperatively and postoperatively compared with the asymptomatic side may be attributed to pain-related disuse atrophy prior to surgery that could not be fully reversed through postoperative rehabilitation. In contrast, the G. medius/G. minimus complex achieved CSAs comparable to both preoperative values and the asymptomatic side at the 1-year follow-up. This finding may be explained by the absence of a significant preoperative difference between sides, suggesting that this muscle complex may not have been substantially affected by pain-related disuse. Furthermore, the more favorable outcomes observed in the G. medius/G. minimus complex compared with those reported in the literature may be related to differences in postoperative rehabilitation protocols.

The R. femoris is an important muscle that plays a key role in hip flexion, abduction, and rotation [19]. However, surprisingly, some previous studies have reported no significant differences in muscle CSA between the symptomatic and asymptomatic sides in the preoperative period [12, 20]. We believe that the lower muscle CSA observed on the operated side compared with the preoperative asymptomatic side is a consequence of pain-related movement restriction on the affected side. Furthermore, we found that the operated side remained inferior to the asymptomatic side even at the 1-year postoperative follow-up. The pattern of change in R. femoris CSA was similar to that observed in the G. maximus. The iliocapsularis muscle, which is located anterior to the hip joint and contributes to anterior hip stability, has been emphasized in previous studies to be hypertrophic in dysplastic hips [21–23]. Given its anatomical location, the iliocapsularis muscle may be susceptible to injury during capsulotomy; however, consistent with the literature, we observed that its CSA returned to preoperative levels at the 1 year follow-up [15]. However, although no significant difference was observed between sides in the preoperative period, a significant postoperative increase on the asymptomatic side resulted in the emergence of a between-side difference. On the other hand, in line with the literature, no difference was observed between the symptomatic and asymptomatic sides in the preoperative A. longus, brevis, and magnus muscle areas [12]. However, at the first postoperative year, a significant difference emerged only in the A. magnus muscle area between the operated side and the asymptomatic side. This significant difference was attributed to a non-significant decrease in the A. magnus area on the asymptomatic side and a non-significant increase on the operated side. The greater A. magnus muscle area on the operated side may be attributed to a greater focus on the operated limb during the rehabilitation process compared with the asymptomatic side.

In the study conducted by Zhu et al., no significant change in the iliopsoas muscle was observed at the 1-year postoperative follow-up [15]. In another study by Malloy et al., no difference in the iliopsoas muscle was reported between the symptomatic and asymptomatic sides [12]. In our study, the absence of a preoperative difference is consistent with the existing literature; however, the significant increase in muscle CSA observed at the 1-year postoperative follow-up differs from previously reported findings. This discrepancy may be attributable to differences in postoperative rehabilitation protocols or to the heterogeneity of surgical procedures applied to patients included in prior studies. The pattern of change observed in the V. lateralis muscle was similar to that of the iliopsoas muscle. While no preoperative difference was detected between sides, a postoperative increase was observed on both sides. Although the lack of a preoperative difference is consistent with the literature, to the best of our knowledge, data regarding postoperative changes in the V. lateralis muscle CSA are not available in the current literature [12]. The absence of a preoperative difference in adductor muscle CSA between the symptomatic and asymptomatic sides is consistent with the existing literature [12]. At the 1-year follow-up, all adductor muscles regained their preoperative muscle CSA. However, while no reduction was observed in the A. longus and A. brevis on the asymptomatic side, a significant decrease was detected in the A. magnus. This finding may be attributable to the asymptomatic side being relatively neglected or insufficiently addressed in the postoperative rehabilitation program.

Yang et al. reported that the increase in the G. maximus muscle area was correlated with an improvement in the mHHS score [14]. In the study by Zhu et al., an increase in the iliocapsularis muscle area was correlated with improvement in the mHHS score at 6 months, whereas no correlation was observed at 1 year [15]. In contrast to previous reports, the findings demonstrated a correlation between the iliocapsularis muscle area at 1 year and postoperative mHHS scores. Moreover, in line with the literature, the increased G. maximus muscle area was correlated with postoperative mHHS scores. Collectively, these results indicate that appropriate rehabilitation may contribute to improved functional outcomes.

The retrospective design of the study and the inability to control for factors such as rehabilitation adherence, activity level, body mass index, and preoperative symptom duration may have influenced postoperative muscle morphology and functional outcomes. Additionally, although previous studies have reported associations between muscle CSA and muscle strength, muscle CSA alone does not directly reflect muscle strength or neuromuscular performance, both of which are influenced by multiple physiological factors [24–26]. Although postoperative MRI evaluation was routinely performed at the 1-year follow-up in our institution, the exclusion of patients with incomplete follow-up may have influenced the study results. Furthermore, although the asymptomatic contralateral hip was used as an internal control to reduce interindividual variability, subclinical femoroacetabular impingement morphology or early pathological changes may have been present despite the absence of clinical symptoms. Nevertheless, this study contributes to the existing literature by providing a comprehensive evaluation of most peri-hip muscle groups on both the symptomatic and asymptomatic sides in the preoperative period and at the 1-year postoperative follow-up. Moreover, the study encompasses the available literature to date and includes the assessment of muscle groups that have not been previously evaluated.

## Conclusion

In hips with femoroacetabular impingement, the muscle areas of the G. maximus and R. femoris are smaller than those of the asymptomatic side and do not reach asymptomatic levels at 1-year postoperatively. In contrast, no differences were observed between the two sides in the G. medius/G. minimus muscle complex either preoperatively or postoperatively. Following hip arthroscopy, increases in G. maximus muscle area, changes in postoperative iliocapsularis muscle area, and postoperative iliocapsularis muscle area itself were found to be potentially associated with mHHS scores.

## Data Availability

The datasets used and/or analyzed during the current study are available from the corresponding author on reasonable request.
